# Validation of the european drug addiction prevention trial questionnaire (EU-DAP) for substance use screening and to assess risk and protective factors among adolescents in chile

**DOI:** 10.1192/j.eurpsy.2021.258

**Published:** 2021-08-13

**Authors:** J. Gaete, S. Ramirez, S. Gana, M. Godoy, M. Valenzuela

**Affiliations:** 1 Faculty Of Education, Universidad de los Andes, Santiago, Chile; 2 Department Of Educational Assessment, Measurement And Registry, Universidad de Chile, Santiago, Chile; 3 Department Of Psychology Invest Research Flagship, University of Turku, Turku, Finland

**Keywords:** Substance use, adolescents, Risk factors, validation

## Abstract

**Introduction:**

Substance use is highly prevalent among Chilean adolescents, and the damage it causes at the neurobiological, psychological, and social levels is well known. However, there are no validated screening instruments that also assess risk and protective factors for this Chilean population.

**Objectives:**

To evaluate the psychometric properties of the European Drug Addiction Prevention Trial Questionnaire (EU-Dap).

**Methods:**

A cross-sectional study was carried out in 13 schools in Santiago of Chile. The sample included 2,261 adolescents of 10 to 14 years old. The linguistic and cultural adaptation was conducted using focus groups, the construct validity was evaluated using confirmatory factor analysis, and measures of its reliability were also determined. Furthermore, the associations regarding risk and protective factors with substance use were explored.

**Results:**

Substance use questions were well understood by adolescents. Regarding the subscales of risk and protective factors, they needed some changes, and once completed, all new subscales had good or adequate goodness of fit adjustment. Regarding reliability, all of the new subscales had good or acceptable internal consistency according to the omega coefficient (range from 0.69 to 0.89). Finally, most of the risk and protective factors measured by the questionnaire were strongly associated with different substance use outcomes, especially those related to positive and negative beliefs or attitudes towards drugs, normative beliefs, and refusal skills.

**Conclusions:**

The current findings suggest that the EU-Dap questionnaire is a valid and reliable instrument, and it may help to evaluate the effectiveness of preventive interventions in the future.

**Disclosure:**

No significant relationships.

